# Around the Hospitals

**Published:** 1893-06-03

**Authors:** 


### AROUND THE HOSPITALS.

NOTICE TO THE MANAGERS OF INSTITUTIONS.—Special space is now reserved for the insertion of notices of hospital meetings and festivals. The Editor requests that all notices may reach THE HOSPITAL Office, 428, Strand, at least one week before the date of each meeting.

**The Chelsea Hospital for Women.**—This hospital possesses some excellent regulations. Whilst really necessitous patients are received for gratuitous treatment through subscribers' letters, those who can contribute to their own maintenance are expected to do so. Payments are from 10s. 6d. to £2 2s. per week. Applicants for admission through letter are entered for admission on the register for admission in rotation when vacancies occur. An arrangement has recently been made whereby three beds have been allotted to each physician to out patients after some years of service. The hospital, which possesses but 60 beds, has great demands made upon its limited space. That visitors are welcome is obvious, since directions through which the hospital may be reached from various points are clearly given. A series of post-graduate lectures are given by the hospital staff. The hospital has adopted the letter-card method of special appeal, which has proved economical and effective. The convalescent home at St. Leonards has proved most successful and beneficial.

**The New Hospital for Women.**—During the past year 500 patients have been received, and 21,866 visits by out patients have been paid. These numbers show an increase on any previous year. The hospital was founded with 10 beds; it now contains 42. A maternity department has been opened, and poor women are now attended in their own homes by a qualified medical woman. The hospital affords an opportunity desired by many women of receiving medical aid from their own sex. It affords invaluable experience to future women practitioners. The reliable income of the institution amounts to £1,600, whilst the income equals about £3,300. The value of the work is shown by the extensions that have proved needful from time to time.

